# Conservation Planning for Offsetting the Impacts of Development: A Case Study of Biodiversity and Renewable Energy in the Mojave Desert

**DOI:** 10.1371/journal.pone.0140226

**Published:** 2015-11-03

**Authors:** Jason Kreitler, Carrie A. Schloss, Oliver Soong, Lee Hannah, Frank W. Davis

**Affiliations:** 1 Western Geographic Science Center, U.S. Geological Survey, Boise, ID, United States of America; 2 Contractor to the Western Geographic Science Center, U.S. Geological Survey, Boise, ID, United States of America; 3 Bren School of Environmental Science and Management, University of California Santa Barbara, Santa Barbara, CA, United States of America; 4 The Betty and Gordon Moore Center for Science and Oceans, Conservation International, Arlington, VA, United States of America; University of Southern California, UNITED STATES

## Abstract

Balancing society’s competing needs of development and conservation requires careful consideration of tradeoffs. Renewable energy development and biodiversity conservation are often considered beneficial environmental goals. The direct footprint and disturbance of renewable energy, however, can displace species’ habitat and negatively impact populations and natural communities if sited without ecological consideration. Offsets have emerged as a potentially useful tool to mitigate residual impacts after trying to avoid, minimize, or restore affected sites. Yet the problem of efficiently designing a set of offset sites becomes increasingly complex where many species or many sites are involved. Spatial conservation prioritization tools are designed to handle this problem, but have seen little application to offset siting and analysis. To address this need we designed an offset siting support tool for the Desert Renewable Energy Conservation Plan (DRECP) of California, and present a case study of hypothetical impacts from solar development in the Western Mojave subsection. We compare two offset scenarios designed to mitigate a hypothetical 15,331 ha derived from proposed utility-scale solar energy development (USSED) projects. The first scenario prioritizes offsets based precisely on impacted features, while the second scenario offsets impacts to maximize biodiversity conservation gains in the region. The two methods only agree on 28% of their prioritized sites and differ in meeting species-specific offset goals. Differences between the two scenarios highlight the importance of clearly specifying choices and priorities for offset siting and mitigation in general. Similarly, the effects of background climate and land use change may lessen the durability or effectiveness of offsets if not considered. Our offset siting support tool was designed specifically for the DRECP area, but with minor code modification could work well in other offset analyses, and could provide continuing support for a potentially innovative mitigation solution to environmental impacts.

## Introduction

The impacts of climate and land use change are some of the largest challenges facing biodiversity conservation and natural resource management in the 21^st^ century. Increasing the amount of renewable energy generation is a primary goal to mitigate emissions contributing to climate change from the energy sector. The direct footprint of utility-scale solar energy development (USSED–facilities with >1MW capacity) is large and has an even greater associated area of disturbance [1, 2]. Therefore, to understand tradeoffs between the positive and negative impacts of USSED, the short and long term ecological effects and environmental risks of these developments need to be carefully considered [3], though little information on risks or effects exists in the scientific literature [1].

The mitigation hierarchy of the U.S. National Environmental Policy Act (NEPA) is a commonly used guide in environmental impact assessment for projects such as USSED. It specifies four types of actions to mitigate environmental impacts in decreasing order of preference—avoid, minimize, restore, and offset [4]. Recent research addressing USSED impact mitigation has focused on each of these actions, including Stoms et al.[5] and Cameron et al. [6], who show USSED can be sited to avoid and minimize areas of ecological value; Hernadez et al.[7] quantify the potential of small scale and USSED within the built environment to avoid impacts; and Moilanen [8] and Kiesecker et al.[9] use conservation planning tools to minimize and then prioritize offset sites to mitigate impacts.

Offsets are a valuable tool to protect or enhance habitat for species or natural communities when impacts cannot be avoided, minimized, or restored, by securing habitat or environmental benefits at unaffected sites similar to those impacted [10, 11]. The goal of offsets is to achieve a net neutral or positive outcome for biodiversity [9, 10]. Many challenges and potential criticisms exist in selecting offset sites, both in the theory and policy realms [12–15], in determining the actual additionality of offset actions [13, 16], and in the practice of locating offset sites [8, 9, 17]. For the latter, a challenge in directly offsetting impacts is to satisfy the mitigation requirements that sites are superior in terms of their characteristics including species representation, ecological condition, continued certainty in the future, connectivity to other sites, and cost-effectiveness. Most of these site characteristics can be measured or modeled, and therefore included in spatial planning or decision support tools and used in conservation planning activities [8, 9, 18–20].

Applying spatial conservation planning software to offset siting is a recent, but growing use of conservation planning tools. For example, Kiesecker et al. [9] described a multi-species framework for selecting the minimum set of offset sites to meet targets for each species impacted using Marxan [21]. Several other studies [6, 17] have used Marxan to select offset sites, and have generally following the procedure outlined in Kiesecker et al.[9].

In this study we develop an offset siting model that has similarities but largely differs from previous offset siting models [9, 17] by using a utility maximization problem [22] as the underlying problem formulation. Our implementation considers multiple development projects and ranks all areas in the region by potential value for conservation offsets [8]. We use the following hypothetical questions to guide offset decision support: which sites or sites could most cost-effectively offset impacts of development? Where should offsets be sited if they are required to remain within a specified geographic region or land ownership type? How do selected sites compare when they are prioritized to maximize the biodiversity conservation gain for the full set of conservation features, as opposed to those directly affected by specific projects?

We explore these issues in a case study in the western Mojave Desert of California. The region has many proposed USSED projects, but contains habitat for a number of endemic and endangered species. This combination of factors leads to an important opportunity for conservation and climate change mitigation: renewable energy goals require an increase in solar energy generation, yet how can that increased capacity minimize its impact on biodiversity persistence in a fragile desert ecosystem?

## Material and Methods

**Fig 1:**
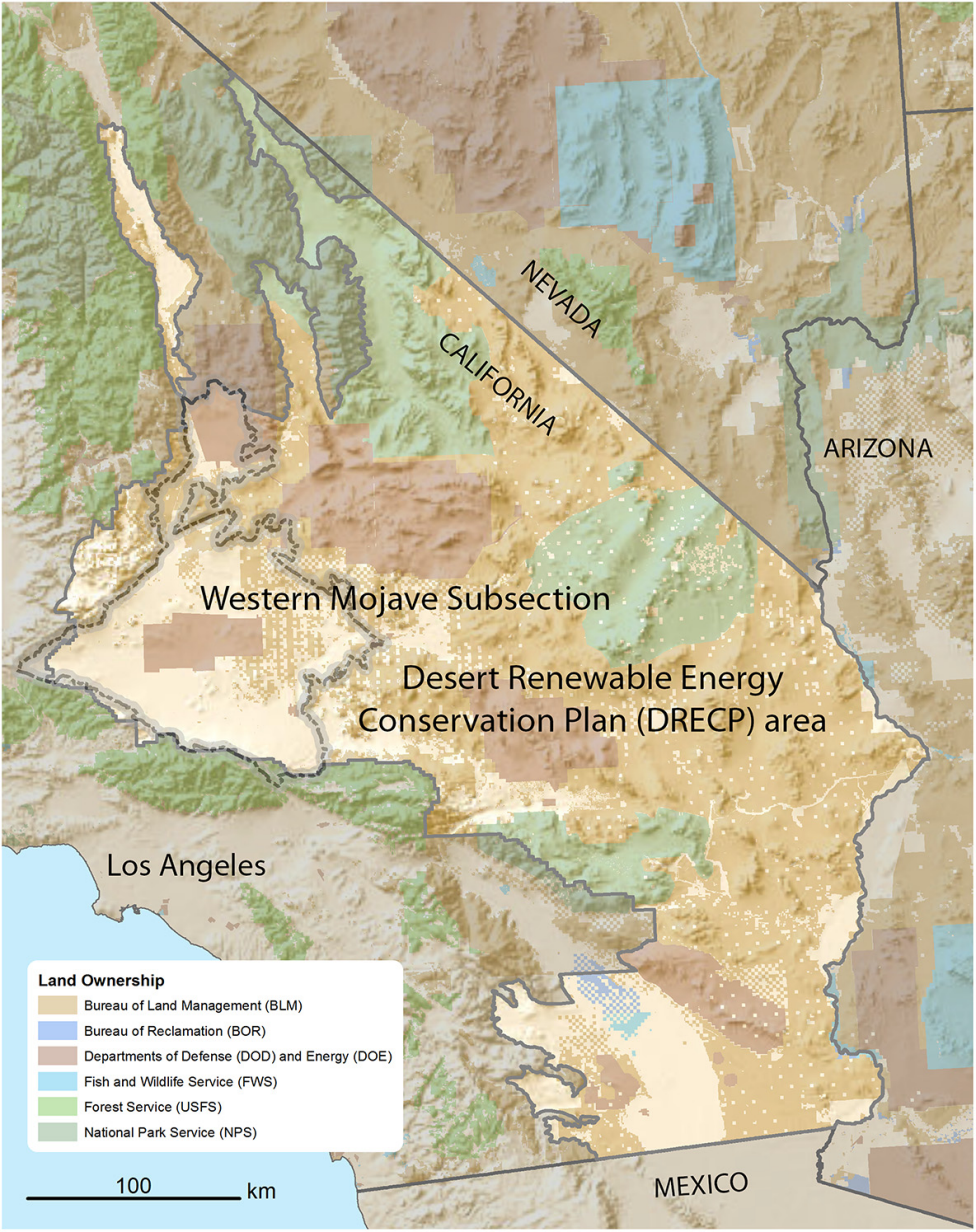
Location map. The Western Mojave DRECP study area (California Ecological Subsection 322Ag: High Desert Plains and Hills [30].The remainder of the methods section is organized as follows: a brief explanation of the Zonation conservation software package [24, 25] precedes further detail describing options of the Mojavset offsetting tool. This is followed by a description of our case study site and offsetting decisions, the creation of our set of hypothetical USSED sites, the spatial data describing conservation features, and a description of the two offset analyses analyzed in the case study.

We developed Mojavset, an offset siting support tool, to evaluate the potential impacts to biodiversity from a solar development site, or set of sites, and to then identify potential biodiversity offset sites for conservation action in the Desert Renewable Energy Conservation Plan area (DRECP, www.drecp.org, Fig 1). The DRECP is a federal and state effort that seeks to simultaneously conserve and manage biodiversity while efficiently guiding the permitting and development of renewable energy in the desert region. Mojavset provides decision support for three steps of the mitigation hierarchy. The primary purpose of the tool is the spatial prioritization of offset sites; the avoid and minimize steps are implemented to alert a user if sites are in direct conflict with incompatible lands or sites of known high conservation priority, before an offset analysis is performed. Restoration, in this DRECP example, is not considered a viable option due to the slow rate of recovery in desert ecosystems [23]. Selected offset sites are presumed protected indefinitely from future development and to retain their desired ecological characteristics. While not implemented in this case study, Mojavset does have an option to incorporate alternative land use scenarios to prioritize offsets in areas expected to be lost without conservation intervention; thus incorporating additionality to ensure ‘no net loss’ [10, 16].

### Zonation background

**Fig 2:**
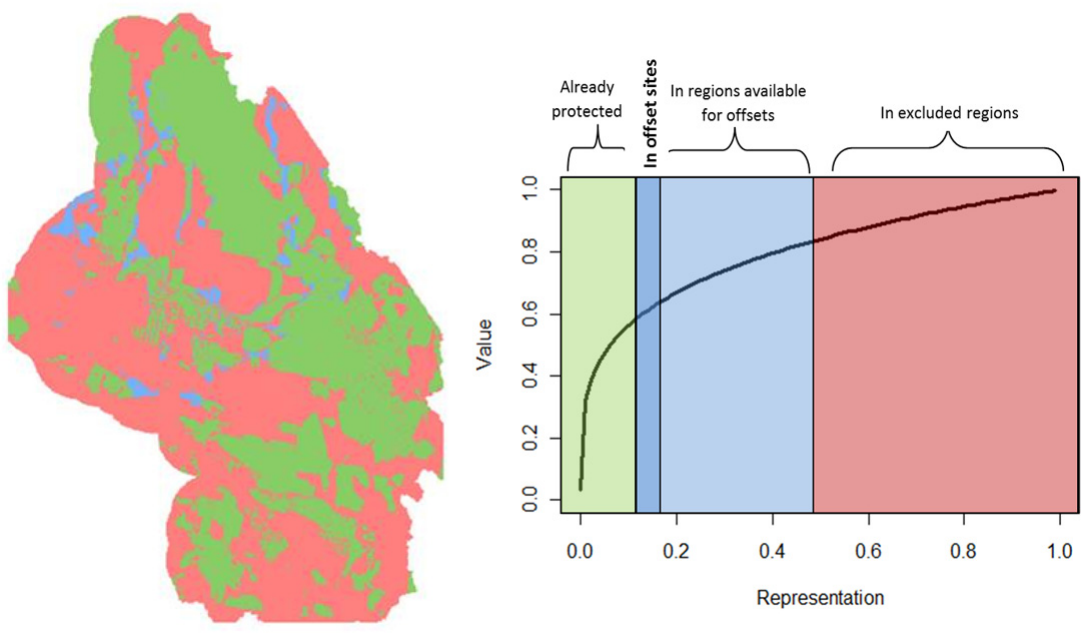
Map illustrating the use of a utility curve in Zonation. The colors on both map and chart correspond to regions of protected lands (green), area available for offsets (blue), selected offset sites (darker blue), and areas excluded for offset site consideration (red). Map is for illustrative purposes and does not accurately reflect current availability status.

Zonation is a conservation planning tool that uses the concept of conservation utility functions [24, 26], and is used extensively in spatial conservation prioritization [24, 27]. The use of utility functions is a primary difference compared to other planning routines [22], and represents the relationship between the amount of a particular conservation feature and its social value. For example, in a utility curve in the DRECP study area, total value increases non-linearly with the degree of protection (representation) of a conservation feature, such that the marginal utility of additional protection or restoration for a particular biological feature diminishes as total protection of that feature across the planning region increases (Fig 2). Zonation allows separate utility curves for each conservation feature and employs an iterative removal algorithm to remove units (planning units or grid cells) from the conservation solution in an order that produces the smallest loss of total conservation value at each step [24]. The order of removal determines the relative value of each cell and results in a hierarchy of conservation value across the landscape.

### Mojavset–an offset siting support tool

Mojavset [28] is a collection of functions for the public R statistics library [29] to process geospatial data, take user inputs, and run the Zonation software to prioritize sites for offsets. Mojavset generates the required Zonation input files via a series of user responses to text prompts. Along with the standard Zonation output, Mojavset generates ASCII grids that delineate potential offset sites and corresponding site reports with information on land management and biodiversity representation. A thorough treatment of each decision point, Zonation option, dataset, and type of offset analysis is included in the Mojavset user manual [28]; an abbreviated explanation is provided below.

Offset analysis begins with choosing to directly offset impacts or to identify offset areas based on a goal of maximizing conservation gains, evaluated based on species distributions, ecological systems, or both. In the case of direct offsets (hereafter Direct), only conservation features that will be impacted by potential solar sites are used for offset prioritization. For example, if 8 species and 3 ecological systems will be impacted, then only these 11 features out of the entire suite of species and ecological systems will be considered when prioritizing sites for offsets. The distributions of all other conservation features will be ignored and will not add value to a network of offsets sites.

To prioritize offsets with the maximum biodiversity conservation gains option (hereafter Max Cons), any mapped conservation feature is considered available for offset prioritization, or a subset of features weighted by importance. Zonation will then prioritize the landscape based on complementarity (core area zonation [24]), and feature richness (additive benefit function [24]). Mojavset prioritizes offset sites that are identified in both planning methods and the areas that are unique to each method. For both Direct and Max Cons planning types, offset target amounts are set based on an offset ratio of the impacted area.

### Case Study of Mojavset for offset siting in the Western Mojave

To demonstrate Mojavset, we evaluated proposed solar projects in the Western Mojave region, as defined by Goudey and Smith [30] (Fig 1). This test case is purely a demonstration; future offset planning and analyses should be vetted through the appropriate processes and channels, with official data, species priorities, and offset requirements.

The Western Mojave ecological subsection is a 1.27 million hectare region in the western region of the DRECP boundary (Fig 1). The region is more populated than other areas within the DRECP planning region, and contains the cities of Lancaster, Palmdale, and Barstow, among others. Local land use authority is divided among three county planning agencies, multiple urban incorporated areas, and state and federal agencies. Much of the region is modified from natural condition by human action, and a high proportion of the area is within Stoms et al.’s ecologically “highly degraded condition” class [5]. The area still contains a high level of native biodiversity, however, and the juxtaposition of a high concentration of proposed solar projects and numerous species covered under the Endangered Species Act, California Endangered Species Act, or listed as sensitive or of special concern [31], makes this a particularly interesting and potentially useful study area to test Mojavset.

### Proposed utility scale solar projects

**Fig 3:**
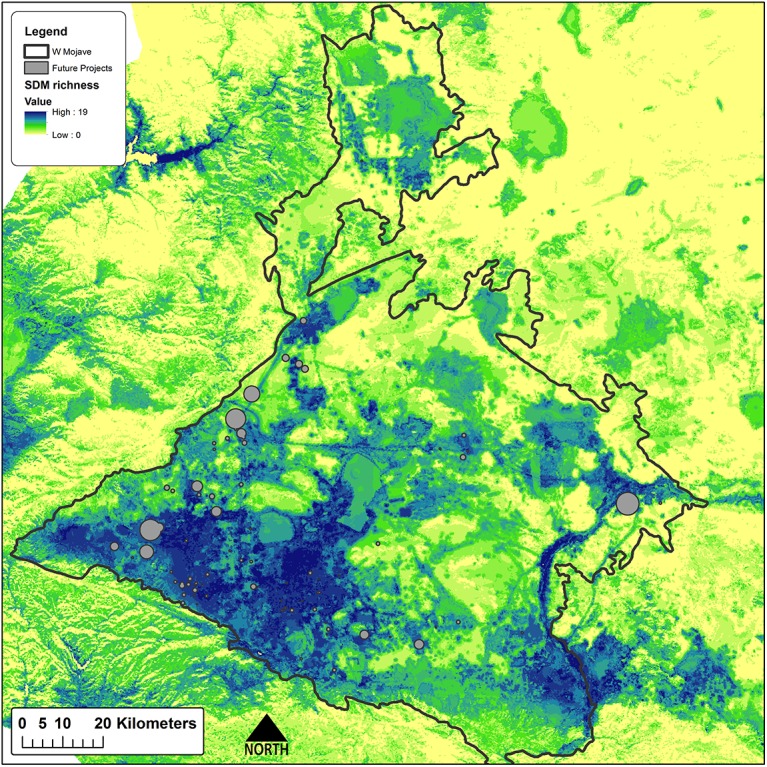
Proposed solar development sites. Western Mojave ecological subsection with species richness and proposed utility scale solar development locations. Species richness is based on SDMs described in Davis et al. [15].

The California Energy Commission maintains information detailing the location and size of existing and proposed utility scale (>1MW) solar facilities (http://www.energy.ca.gov/33by2020/). We queried this database to determine the location and size of potential facilities for our offset analysis and demonstration. 71 USSED projects are proposed within the Western Mojave ecological subsection, with an estimated total capacity of just less than 3500 MW of power and covering 15,331 ha (Table 1, Fig 3). Proposed projects are still in the planning and permitting phases, and actual project boundaries were not available at the time of this study. To approximate their footprint we created circular impact sites with area equal to the reported area, centered on the reported coordinates. This approximation likely captures the scale of impacts from the proposed projects, but will not represent the final impacts with the accuracy required for mitigation. Furthermore, our footprint approximation will not fully represent the actual cumulative impacts of all aspects of USSED activities [1, 2, 32].

**Table 1:** Impact site characteristics from the hypothetical proposed projects.

Area of Sites (ha)	15,331
Ownership (max area)	Department of Defense
% Federal Ownership	2
USGS GAP Status–area majority	4
USGS GAP Status–minimum status	3
% Overlap with TNC priority	9
% Overlap with BLM Areas of Critical Environmental Concern	0
# of Targeted Species	33
% of Targeted Species	51
Maximum of Range for a Target Species (%)	1
# of Ecological Systems	14
% of Ecological Systems	15
Maximum of Range for an Ecological System (%)	1
Mean Slope (%)	2
Mean Direct Normal Irradiance (DNI: kWh/m^2^/day)	8

### Conservation features

For this test case, we considered 64 plant and animal species (Table 2), chosen based on guidance from the Independent Science Advisers Report [31]. Feature data consisted of species distribution models at 270 m resolution, modeled with Maxent [33] according to current best practices and procedures [34–37]. Further detail on the species distribution modeling can be found in Davis et al. [38].

**Table 2:** Species list, summary of modeled solar development impact, and offsets achieved for each species based on Direct vs. Max Cons objectives.

Species	Common name	Impact	SDM	Target	Direct	Achieved	Max cons	Achieved
		Site (ha)	Total (ha)	(ha)	offsets (ha)	(%)	offsets (ha)	(%)
*Chaetodipus fallax pallidus*	San Diego pocket mouse	139	16,68,142	277	5,169	1866%	5,672	2047%
*Taxidea taxus*	American badger	14,296	31,14,886	28,591	28,679	100%	29,831	104%
*Xerospermophilus mohavensis*	Mohave ground squirrel	9,083	14,63,263	18,167	21,250	117%	21,345	117%
*Agelaius tricolor*	Tricolored blackbird	9,426	15,37,308	18,852	18,969	101%	11,278	60%
*Asio otus*	Long-eared owl	10,345	27,26,752	20,689	21,134	102%	14,981	72%
*Athene cunicularia*	Burrowing owl	11,540	12,97,438	23,080	23,146	100%	14,062	61%
*Buteo regalis*	Ferruginous hawk	9,630	23,32,990	19,260	20,791	108%	18,677	97%
*Buteo swainsoni*	Swainson's hawk	9,739	23,58,949	19,479	19,596	101%	16,118	83%
*Empidonax traillii extimus*	southwestern willow flycatcher	131	7,54,493	262	2,267	864%	824	314%
*Falco columbarius*	Merlin	7,990	24,81,800	15,980	16,082	101%	13,122	82%
*Falco mexicanus*	Prairie falcon	12,502	26,58,240	25,005	27,206	109%	25,974	104%
*Lanius ludovicianus*	loggerhead shrike	11,737	41,41,996	23,474	30,086	128%	29,058	124%
*Laterallus jamaicensis coturniculus*	California black rail	0	5,29,086	0	0	NA	0	NA
*Melanerpes uropygialis*	Gila woodpecker	0	7,14,325	0	0	NA	0	NA
*Toxostoma bendirei*	Bendire's thrasher	7,523	15,69,173	15,047	15,105	100%	9,273	62%
*Toxostoma lecontei*	Le Conte's thrasher	9,696	31,26,185	19,391	26,361	136%	28,103	145%
*Vireo bellii pusillus*	Least Bell's Vireo	139	7,00,934	277	2,741	989%	1,137	411%
*Charina trivirgata*	rosy boa	0	8,24,922	0	423	NA	204	NA
*Crotalus ruber*	red-diamond rattlesnake	0	6,54,387	0	459	NA	1,188	NA
*Phrynosoma blainvillii*	coast horned lizard	926	14,33,447	1,852	4,855	262%	3,339	180%
*Phrynosoma mcallii*	Flat-tail horned lizard	0	4,22,259	0	0	NA	0	NA
*Uma scoparia*	Mojave fringe-toed lizard	87	2,43,078	175	190	108%	44	25%
*Abronia villosa var aurita*	desert sand verbena	117	9,51,068	233	2,151	922%	1,728	741%
*Acmispon argyraeus var multicaulis*	Scrub lotus	36	77,733	73	87	120%	211	290%
*Allium nevadense*	Nevada onion	0	2,35,963	0	0	NA	0	NA
*Androstephium breviflorum*	pink funnel lily	66	2,11,403	131	146	111%	0	0%
*Arctomecon merriamii*	desert poppy	124	3,67,146	248	598	241%	22	9%
*Asclepias nyctaginifolia*	Mojave milkweed	0	1,53,535	0	0	NA	0	NA
*Astragalus cimae var cimae*	Cima milk vetch	0	88,173	0	0	NA	0	NA
*Astragalus insularis var harwoodii*	Harwood's milk vetch	0	3,12,588	0	0	NA	0	NA
*Astrolepis cochisensis ssp cochisensis*	scaly cloak fern	0	3,10,299	0	95	NA	22	NA
*Boechera shockleyi*	Shockley's rock cress	0	2,98,788	0	0	NA	7	NA
*Calochortus striatus*	alkali mariposa lily	2,610	2,06,803	5,220	10,024	192%	23,109	443%
*Castela emoryi*	Cricifixion Thorn	7	4,14,998	15	175	1200%	7	50%
*Chorizanthe parryi var parryi*	Parry's spineflower	0	1,26,124	0	15	NA	44	NA
*Cordylanthus parviflorus*	small-flowered bird's beak	0	81,488	0	0	NA	0	NA
*Coryphantha alversonii*	Alverson's foxtail cactus	0	5,54,361	0	0	NA	0	NA
*Coryphantha chlorantha*	desert pincushion	0	1,25,067	0	0	NA	0	NA
*Cymopterus deserticola*	desert cymopterus	467	2,31,428	933	8,413	902%	18,903	2026%
*Cymopterus gilmanii*	Gilman's cymopterus	0	3,19,630	0	0	NA	0	NA
*Cymopterus multinervatus*	purple-nerve cymopterus	109	1,78,299	219	219	100%	15	7%
*Delphinium recurvatum*	recurved larkspur	15	5,001	29	350	1200%	1,524	5225%
*Enneapogon desvauxii*	nine-awned pappus grass	0	2,41,146	0	0	NA	0	NA
*Eriastrum harwoodii*	Harwood's eriastrum	0	4,92,177	0	0	NA	0	NA
*Erioneuron pilosum*	hairy erioneuron	0	2,63,322	0	0	NA	0	NA
*Eriophyllum mohavense*	Barstow woolly sunflower	642	2,68,192	1,283	7,880	614%	12,969	1011%
*Eschscholzia minutiflora ssp twisselmannii*	Red Rock poppy	131	1,74,588	262	1,123	428%	1,254	478%
*Layia heterotricha*	pale-yellow layia	0	46,292	0	365	NA	2,027	NA
*Mimulus mohavensis*	Mojave monkeyflower	1,290	2,34,913	2,581	2,661	103%	204	8%
*Monardella robisonii*	Robison's monardella	0	1,99,061	0	0	NA	0	NA
*Muhlenbergia appressa*	appressed muhly	0	4,35,242	0	0	NA	0	NA
*Opuntia basilaris var treleasei*	Bakersfield cactus	277	1,17,631	554	3,324	600%	5,059	913%
*Pellaea truncata*	spiny cliff-brake	0	1,31,402	0	0	NA	0	NA
*Penstemon albomarginatus*	white-margined beardtongue	0	47,480	0	0	NA	0	NA
*Penstemon stephensii*	Stephens' beardtongue	0	1,46,026	0	0	NA	0	NA
*Penstemon utahensis*	Utah beardtongue	0	56,046	0	0	NA	0	NA
*Phacelia nashiana*	Charlotte's phacelia	219	2,67,368	437	1,582	362%	773	177%
*Psorothamnus fremontii var attenuatus*	narrow-leaved psorothamnus	0	1,42,177	0	0	NA	0	NA
*Sanvitalia abertii*	Abert's sanvitalia	0	96,877	0	7	NA	7	NA
*Senna covesii*	Cove's cassia	0	3,81,077	0	29	NA	0	NA
*Sphaeralcea rusbyi var eremicola*	Rusby's desert-mallow	0	1,65,053	0	0	NA	0	NA
*Stipa arida*	Mormon needle grass	7	7,04,753	15	73	500%	0	0%
*Symphyotrichum defoliatum*	San Bernardino aster	0	64,254	0	0	NA	0	NA
*Yucca brevifolia*	Joshua tree	386	14,37,486	773	6,102	790%	5,919	766%

### Case study scenarios

We compared two offset scenarios in this case study, illustrating two types of offset analyses in Mojavset: direct offsets for the impacted features, and a Max Cons scenario that identifies priorities for both impacted and non-impacted features using the additive benefit function. We used a 2:1 offset ratio (2 offset units per unit of development), and ecological condition [5] as a proxy for site cost (the lower the ecological condition the higher the offset site cost). Locations of proposed utility scale solar facilities provided the set of hypothetical impact sites, and the Western Mojave subsection served as an analysis mask to specify the planning region. Offset sites were allowed on private and public lands, except public lands designated with permanent biodiversity protection (GAP 1 and 2 status public lands).

### Results: Projected Impacts and Offset Scenarios

The hypothetical impact sites of the Western Mojave are distributed primarily in the western portion of the subsection. These projects occur almost exclusively on private land with minimal overlap with priority areas of the Bureau of Land Management (BLM) and The Nature Conservancy (TNC) (Table 1). The sites do tend to occur in areas of greater species richness based on the modeled species distributions (Fig 3), and intersect with the ranges of 33 species of potential conservation concern [31]. The amount of impacted distribution by species and the species’ total modeled distribution is shown in Table 2, and varies from no impact to 14,296 ha, or nearly 93% of the impact area falling within the distribution of the American badger’s (*Taxidea taxis*) range in this study area. The alkali mariposa lily (*Calochortus striatus*) is impacted most as a proportion of its total modeled distribution, at 1.3%.

**Fig 4:**
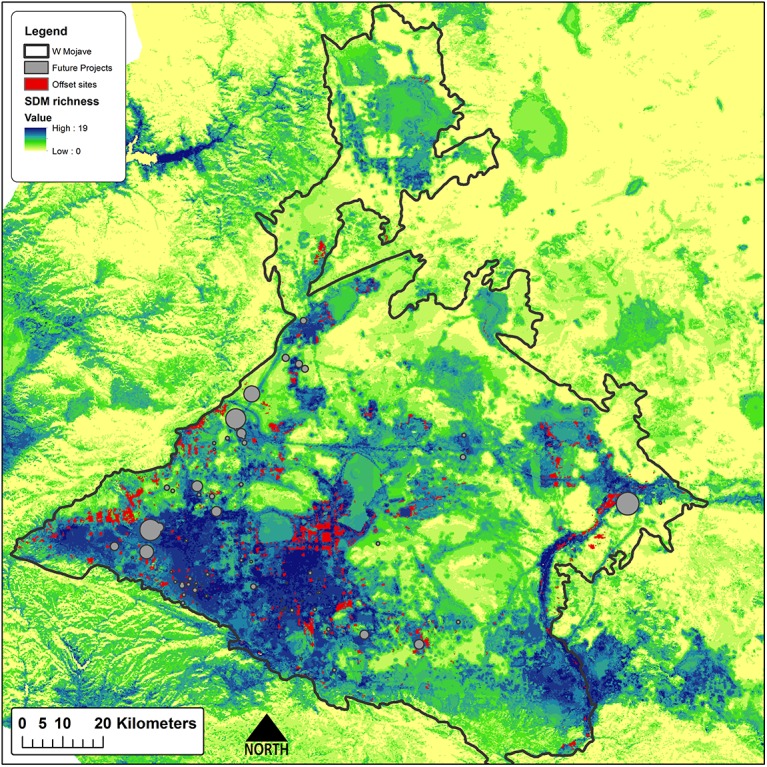
Direct offset sites. Direct offset sites (red) for hypothetical solar energy projects (gray) using a 2:1 offset ratio, over modeled species richness.

**Fig 5:**
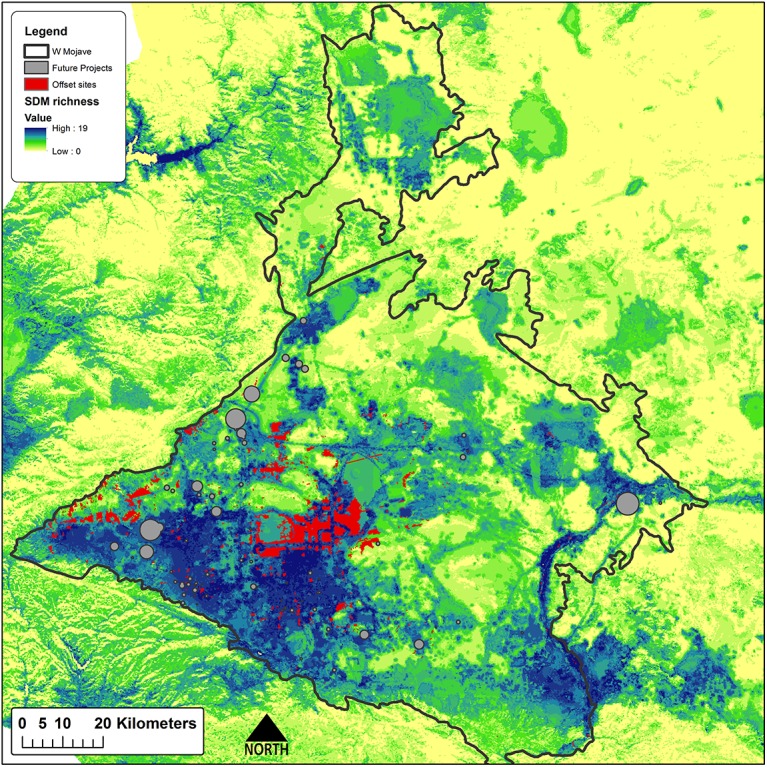
Max Cons offset sites. Max Cons offset sites (red) for hypothetical solar energy projects (gray) using a 2:1 offset ratio, over modeled species richness.

**Fig 6:**
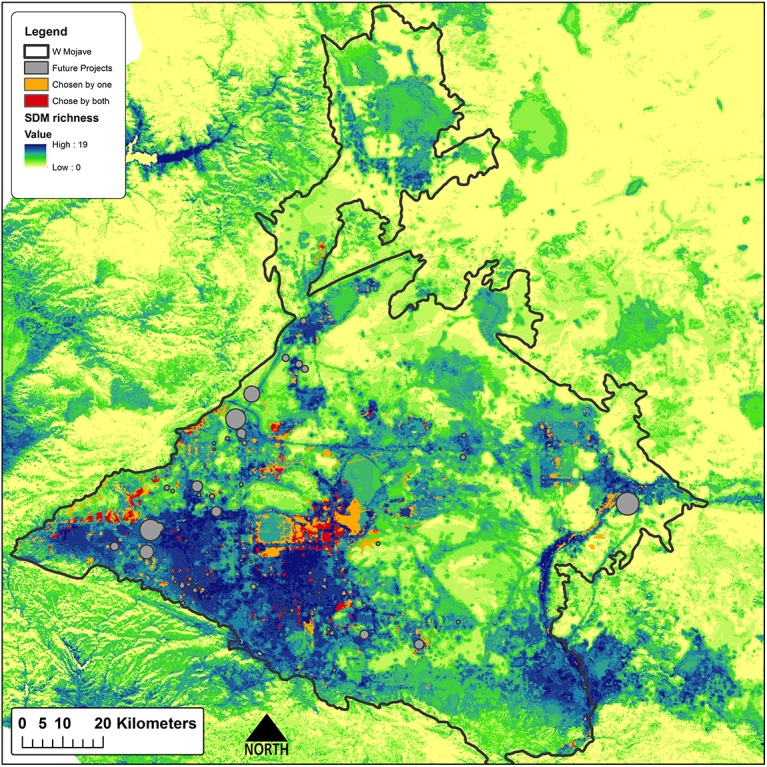
Scenario agreement. The agreement of both scenarios (red), areas chosen by one scenario (orange), over modeled species richness.

Offsets totaling 32,178 ha are required to meet the stated targets of impacted features (Table 3). Sites selected to offset impacts to species by the proposed projects are clustered in species-rich areas in the center of the study region that are in good ecological condition, in areas at the western margin of the study region that support intact habitats for a few species associated with that portion of the study area, and in the eastern and northern portions of the study region where sites provide the most ecologically intact opportunities for some riparian species and narrowly endemic plant species (Fig 4). The important result is that the value of these sites is readily apparent in terms of their composition, condition, land ownership and land management. Sites selected through the Max Cons scenario are more concentrated in species-rich areas in the center of the study region (Fig 5). While not unexpected, this result serves to emphasize that the location of offsets can be sensitive to one or a few individual species and the methods used to prioritize offset sites. The two scenarios did show some level of agreement and overlapped 28% (13,960 ha), primarily in the western and central portions of the study area (Fig 6), indicating their importance for offsets regardless of offset planning type.

**Table 3:** Area (ha) in various land status classes for the Max Cons and Direct offset scenarios.

	Max Cons (ha)	Direct (ha)
Total	32,178	32,178
Federal	18,764	9,501
Department of Defense	18,382	7,792
Bureau of Land Management	309	1,638
Forest Service	73	71
GAP Status 1	0	0
GAP Status 2	6	117
GAP Status 3	577	2,136
The Nature Conservancy (priority conservation lands)	20,781	18,034
Bureau of Land Management (Area of Critical Environmental Concern)	72	1,860

Differences between the two offset scenarios are quite apparent, despite prioritizing the same total area of offsets. The Max Cons scenario sited offsets largely within The Nature Conservancy’s priority habitats, and primarily on Department of Defense (DOD) lands (Table 3). The Direct scenario met all the offset targets and also sited offsets on DOD lands as the majority land owner, but public lands represented less than 30% of that scenario’s offsets (Table 3). Comparing the species results, the Max Cons scenario failed to meet offset targets 14 times (of 33 total species affected), and secured area (3499 ha) for seven species that did not require offset mitigation (Table 2), while the Direct Offset scenario secured more targeted species’ habitat overall (329,924 ha vs 318,034 ha) and secured more habitat in 25 target species cases (216,412 ha vs 166,781 ha) compared to the Max Cons scenario on a species by species basis (Table 2).

## Discussion

Conservation planning tools like Mojavset are designed to identify efficient solutions to problems with a large number of choices. When considering the collective impacts of multiple projects on multiple species, more efficient solutions will likely arise compared to implementing offsets one species at a time on a project-by-project basis. The degree of efficiency will depend on the extent to which modeled species habitats coincide. Determining *a priori* the area required to mitigate unavoidable impacts of multiple projects for many conservation features could potentially assist both energy developers and permit granters to relocate potential site locations to reduce both the potential impact and the cost to offset those impacts.

In our case study of the Western Mojave, the project sites and impacted area are speculative, but provide a good illustration of our offset siting process. A more nuanced approach would simultaneously site solar facilities to meet solar criteria and to minimize impacts. Any residual impacts could be offset with Mojavset, and would likely result in a much smaller area requiring offset mitigation. Previous work in the Mojave has shown there is room to develop utility scale solar with minimal impacts [5, 6], though our use of species level data, as opposed to ecological systems and coarse priority area designations, provides insight into offset siting solutions with greater conservation feature resolution than the aforementioned work.

The efficiency of locating a multi-species offset solution is apparent by comparing the offset area required, given constraints, to the absolute minimum area needed if species were perfectly collocated. With a 2:1 offset ratio and 15,331 ha of impact, the minimum area required for offsets would be 30,662 ha. Thus, the 32,178 ha of the Direct Offsets solution is only 4.9% more area than the hypothetical minimum needed to meet the targets. In other cases where the range of impacted species are more widely distributed and non-overlapping, the total area required to meet targets may be considerably larger than the obligatory minimum area set by the offset ratio. Minimizing the cost or area required to satisfactorily meet mitigation targets is likely the largest benefit of using tools like Mojavset for offset siting.

One interesting result in the case study was the difference between the Direct and Max Cons solutions. The Max Cons solution prioritized the same number of hectares, but only overlapped with the Direct offset solution by 28%, and failed to meet targets for 14 species. This can be explained by the difference in planning method: the Direct scenario had specific species targets to meet, while the Max Cons scenario maximizes overall conservation priority and richness in the region. In practice, all species would require individual weightings and careful consideration of species specific utility functions to ensure the model is parameterized according to users’ goals, and would likely yield a solution more similar to the Direct scenario. This comparison relates to the notion of “strict equivalency” in offset mitigation, or whether offsets can be relaxed to prioritize out of kind targets (targets not directly affected by the actions being offset) [17, 39]. Habib et al. [17] suggest a mechanism for estimating tradeoffs among species or conservation targets, such that any resultant impacts could be mitigated via conservation actions of a higher priority, and claim additional cost savings as a benefit. In theory such a system could align conservation priorities with resources more efficiently than direct offsets. Increasing offset ratios as a function of conservation feature importance and uncertainty could also further discourage impacts and likely produce more certain beneficial outcomes [40]. Other conservation targets, such as important connectivity areas or future species distributions, could also be used as features for impact sites to avoid.

If conservation resources are allocated to offsets, they need to be effective in the long-term to avoid net loss of habitat and increased extinction risk. The literature on design of offsets for climate change is young and emerging, but suggests that offsets could be an effective conservation strategy in response to energy development, *when* species needs in multiple life history stages and across their full range are properly considered [41]. Research on protected area planning for climate change, adaptation to climate change, and assessment of species response to climate change all offer insights that are transportable to offset design [9].

One approach to address offset siting with climate change impacts would give preference to areas that could play an important role in buffering climate change effects [42, 43], and areas expected to be more stable in the face of climate change [44, 45] (e.g., refugia or stable range). An alternative is to conserve portions of the present range of the species, portions of its future range and all intervening connecting suitable habitat to allow the species to move from its present range to future suitable conditions [46]. This latter option presents a much more complex planning problem (even for a single species) and always carries much higher uncertainty.

Higher mitigation offset ratios are also commonly used to cope with uncertainty and to ensure the future durability of offsets [40], and are a relevant adjustment for incorporating climate change concerns into offset planning [41, 47]. Due to the high levels of uncertainty associated with climate change impact projections, very large ratios may be required. Incorporating climate change into the offset planning process could potentially reduce area requirements associated with offset ratios.

A final issue with offsetting as a mitigation tool is ensuring the actions taken are additional to what would have occurred without conservation intervention [8, 13], particularly when restoration is not a viable option [48, 49]. If mitigation funds go towards offset areas that currently support conservation targets, do the offset actions provide a net positive ecological benefit? What if conservation targets are expected to be lost? Methods for estimating potential additionality from offsets are currently being defined [16, 40, 50, 51], but could be modeled by comparing the differences among present and multiple future land use change scenarios. By locating offsets in zones expected to be developed and where biodiversity is threatened in the absence of conservation actions, offsets could meet the goal of no net loss, even if an overall decline in species representation would likely occur [39, 51].

Within the land use policy realm and in applied use, offsetting the impacts of USSED, and development in general, is likely to increase as a mechanism to mitigate the impacts from development and land use change [52]. Mojavset was designed to support joint planning for solar energy and biodiversity conservation, in an effort to increase efficiency and effectiveness of both. With relatively little effort, future work could take the existing Mojavset code and make it portable for other locations. Based on the case study described here, the approach is promising as a tool for planners seeking to balance renewable energy development and biodiversity conservation in the DRECP planning region and beyond.
